# HDAC6 inhibition prevents TNF-α-induced caspase 3 activation in lung endothelial cell and maintains cell-cell junctions

**DOI:** 10.18632/oncotarget.10591

**Published:** 2016-07-13

**Authors:** Jinyan Yu, Mengshi Ma, Zhongsen Ma, Jian Fu

**Affiliations:** ^1^ Department of Respiratory Medicine, The Second Hospital of Jilin University, Changchun, Jilin, P.R. China; ^2^ Center for Research on Environmental Disease, College of Medicine, University of Kentucky, Lexington, KY, USA; ^3^ Department of Toxicology and Cancer Biology, College of Medicine, University of Kentucky, Lexington, KY, USA

**Keywords:** caspase, endothelial, HDAC6, barrier function, inflammation

## Abstract

Pro-inflammatory mediators such as TNF-α induce caspase activation in endothelial cells, which leads to degradation of cellular proteins, induction of apoptotic signaling, and endothelial cell dysfunction. New therapeutic agents that can inhibit caspase activation may provide protection against inflammatory injury to endothelial cells. In the present study, we examined the effects of selective histone deacetylase 6 (HDAC6) inhibition on TNF-α induced caspase 3 activation and cell-cell junction dysfunction in lung endothelial cells. We also assessed the protective effects of HDAC6 inhibition against lung inflammatory injury in a mouse model of endotoxemia. We demonstrated that selective HDAC6 inhibition or knockdown of HDAC6 expression was able to prevent caspase 3 activation in lung endothelial cells and maintain lung endothelial cell-cell junctions. Mice pre-treated with HDAC6 inhibitors exhibited decreased endotoxin-induced caspase 3 activation and reduced lung vascular injury as indicated by the retention of cell-cell junction protein VE-Cadherin level and alleviated lung edema. Collectively, our data suggest that HDAC6 inhibition is a potent therapeutic strategy against inflammatory injury to endothelial cells.

## INTRODUCTION

Inflammation-mediated endothelial cell damage including inflammatory lung vascular injury is often associated with caspase activation and endothelial cell barrier dysfunction [1]. Over-production of pro-inflammatory mediators such as tumor necrosis factor (TNF)-α is a major cause of endothelial cell injury during inflammation [1]. Tumor necrosis factor (TNF)-α-induced caspase activation and endothelial cell dysfunction contribute to inflammatory vascular injury in endotoxemia and sepsis [1]. New agents that can prevent inflammatory injury to endothelial cells could provide therapeutic benefits.

Activated caspases are known to induce apoptosis by cleaving endogenous substrates and alter other cellular functions including cell-cell junctions [2–4]. Caspase-3 activation plays an important role in endothelial cell barrier dysfunction as a result of apoptotic signaling and re-organization of cell-cell junction proteins including ZO-1 and VE-Cadherin [2–4]. Maintaining of endothelial barrier integrity requires interactions of intercellular junctions, actin cytoskeleton and microtubules [5]. Adherens junctions (AJs) and tight junctions play essential roles in supporting endothelial barrier function [2–4]. ZO-1 and VE-cadherin are main components of TJs and AJs respectively. TNF-α induces endothelial barrier disruption by dis-organization of microtubule, actin cytoskeleton and cell junctions [6–9].

HDAC6, a member of class II histone deacetylase (HDACs) [10–12], is localized in the cytoplasm and the nucleus [10–12]. HDAC6 inhibition possesses anti-tumor and anti-inflammation effects [13–15]. HDAC6 has many endogenous substrates including chaperones and cytoskeletal proteins [16]. Stabilization of cytoskeletal proteins and modulation of chaperones by HDAC6 may protect against endothelial cell injury [16, 17]. Indeed, HDAC6 deletion renders mice less vulnerable to endotoxin-induced inflammatory injury [18]. However, the effects of HDAC6 inhibition on caspase 3 activation and endothelial cell function remain to be determined.

Tubastatin A and CAY10603 are potent and highly selective HDAC6 inhibitors with IC50 of 15 nM and 2 pM in a cell-free assay, respectively [19]. Tubastatin A has been reported to possess anti-inflammatory effects [20–23]. Tubastatin A treatment can prevent stress responses and prolong survival during systemic inflammation [24–28].

In the present study, we investigated the effects of HDAC6 knockdown and HDAC6 inhibitors Tubastatin A and CAY10603 on TNF-α-induced caspase 3 activation in endothelial cells *in vitro*. We examined the effects of Tubastatin A and CAY10603 on cell-cell junction integrity and endothelial permeability in primary human lung endothelial cells. Furthermore, the effects of Tubastatin A and CAY10603 on caspase 3 activation and lung edema formation *in vivo* was assessed in a mouse model of endotoxemia.

## RESULTS

### Tubstatin a inhibits TNFα-induced caspase-3 activation in lung endothelial cells

**Figure 1:**
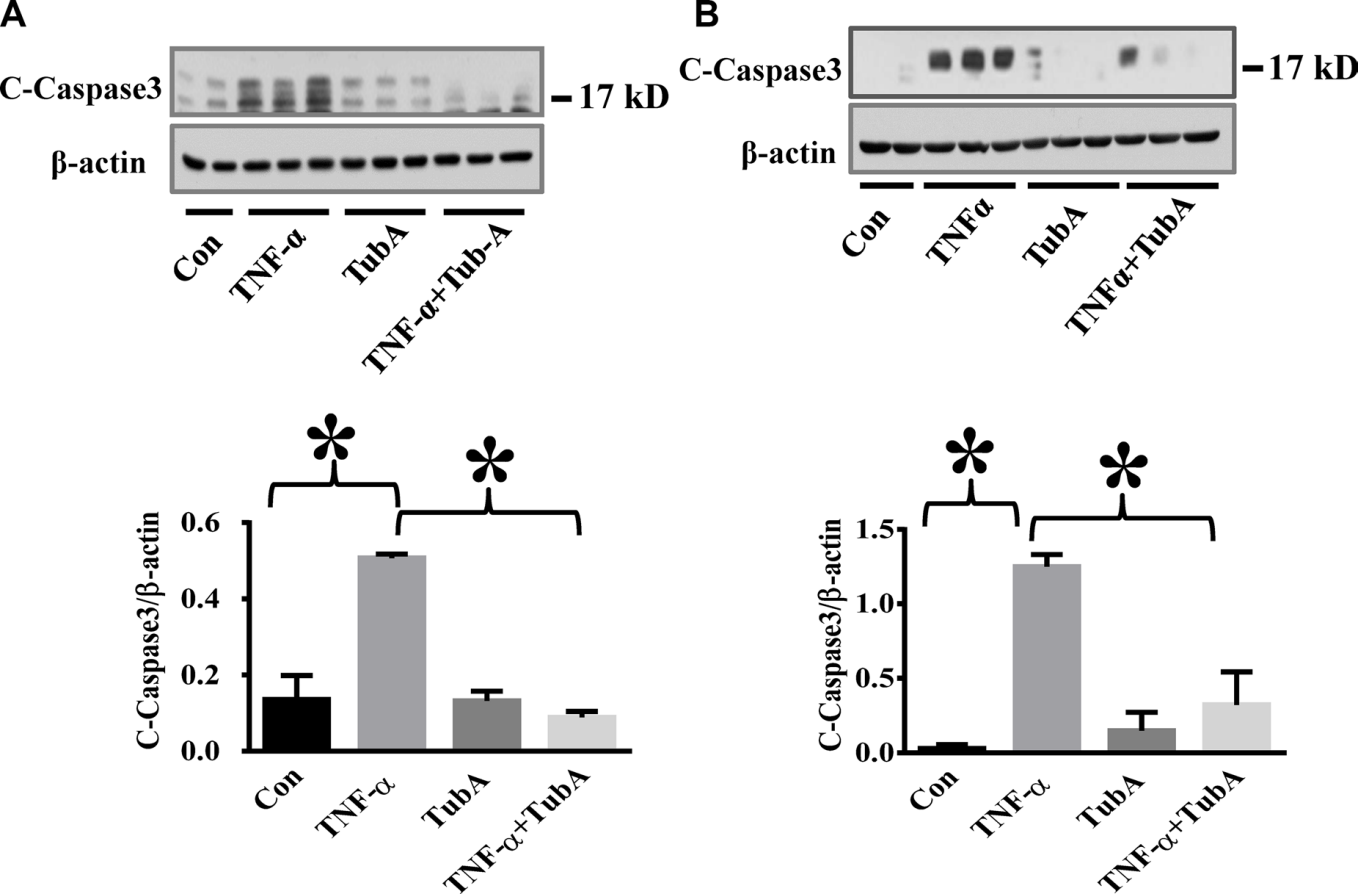
HDAC6 inhibition by Tubastatin A attenuates caspase-3 activation in endothelial cells HPAECs and HLMVECs were pre-treated with Tubastatin A (TubA, 3 μM) for 6 h, then challenged with TNF-α (20 ng/ml) for 18 h. Cells were divided into 4 groups: Control (Con), TNF-α alone (TNFα), Tubastatin A alone (TubA), and TNF-α+Tubastatin A (TNFα+TubA). Representative blots and densitometry analysis of cleaved-caspase-3 in HPAECs (**A**) and HLMVECs (**B**). *^P^ < 0.05 versus TNF-α group, *n* = 3.

TNF-α is a major pro-inflammatory mediator known to induce endothelial injury during inflammation (17, 21, 27). TNF-α induces caspase 3 activation which leads to subsequent cellular damage including endothelial barrier dysfunction [29, 30]. We first assessed whether HDAC6 inhibition can block caspase-3 activation in lung endothelial cells. HPAECs and HLMVECs were pre-treated with Tubstatin A, then challenged with TNF-α. Our results showed that Tubstatin A potently blocked TNF-α-induced caspase-3 activation (cleaved caspase-3 level) in endothelial cells (Figure 1).

### HDAC6 knockdown and CAY10603 treatment attenuate TNF-α-induced caspase-3 activation in endothelial cells

**Figure 2:**
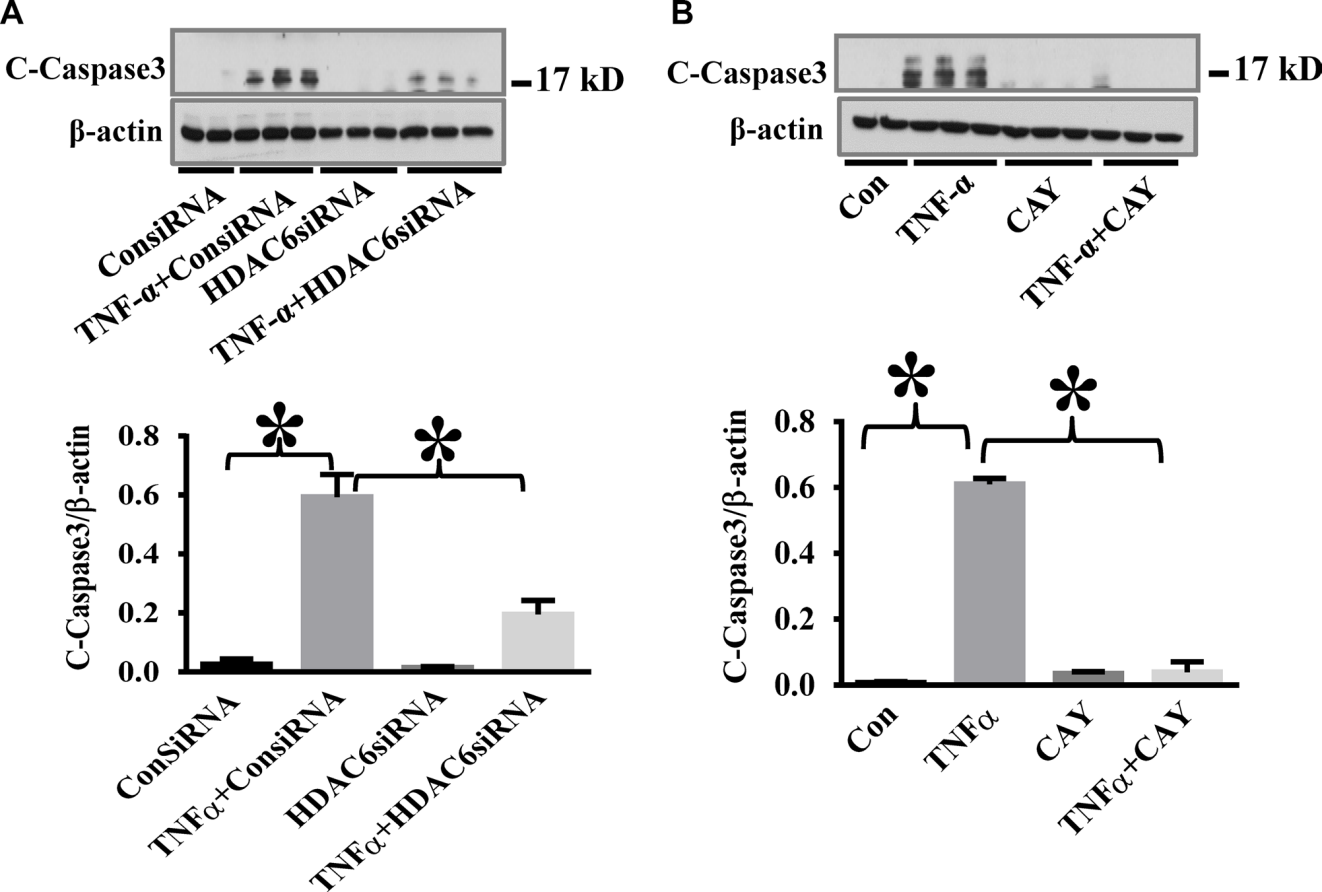
HDAC6 knockdown and HDAC6 inhibition by CAY10603 block TNF-α-induced caspase-3 activation in endothelial cells (**A**) HPAECs were transfected with HDAC6 siRNA or control siRNA for 48 h, then challenged with TNF-α (20 ng/ml) for 24 h. Cells were divided into 4 groups: Control (Con), TNF-α alone (TNFα), siRNA alone (siRNA), and TNF-α+siRNA(TNFα+siRNA). (**B**) HPAECs were pre-treated with CAY10603 (CAY, 0.1 μM) for 6 h, then challenged with TNFα (20 ng/ml) for 18 h. Cells were divided into 4 groups: Control (Con), TNF-α alone (TNFα), CAY10603 alone (CAY), and TNF-α+CAY10603 (TNFα+CAY). Representative blots and densitometry analysis of cleaved caspase-3. *P < 0.05 versus TNF-α group, *n* = 3.

To confirm the specific effects of HDAC6 inhibition on TNF-α-induced caspase-3 activation, we used HDAC6 siRNA knockdown and another selective HDAC6 inhibitor CAY10603 in our study. HPAECs were challenged with TNF-α after siRNA knockdown or CAY10603 treatment. HDAC6 knockdown and CAY10603 treatment both inhibited TNF-α-induced caspase-3 activation (Figure 2).

### HDAC6 inhibition alleviates TNF-α-induced disruption of tight junctions in endothelial cells

**Figure 3:**
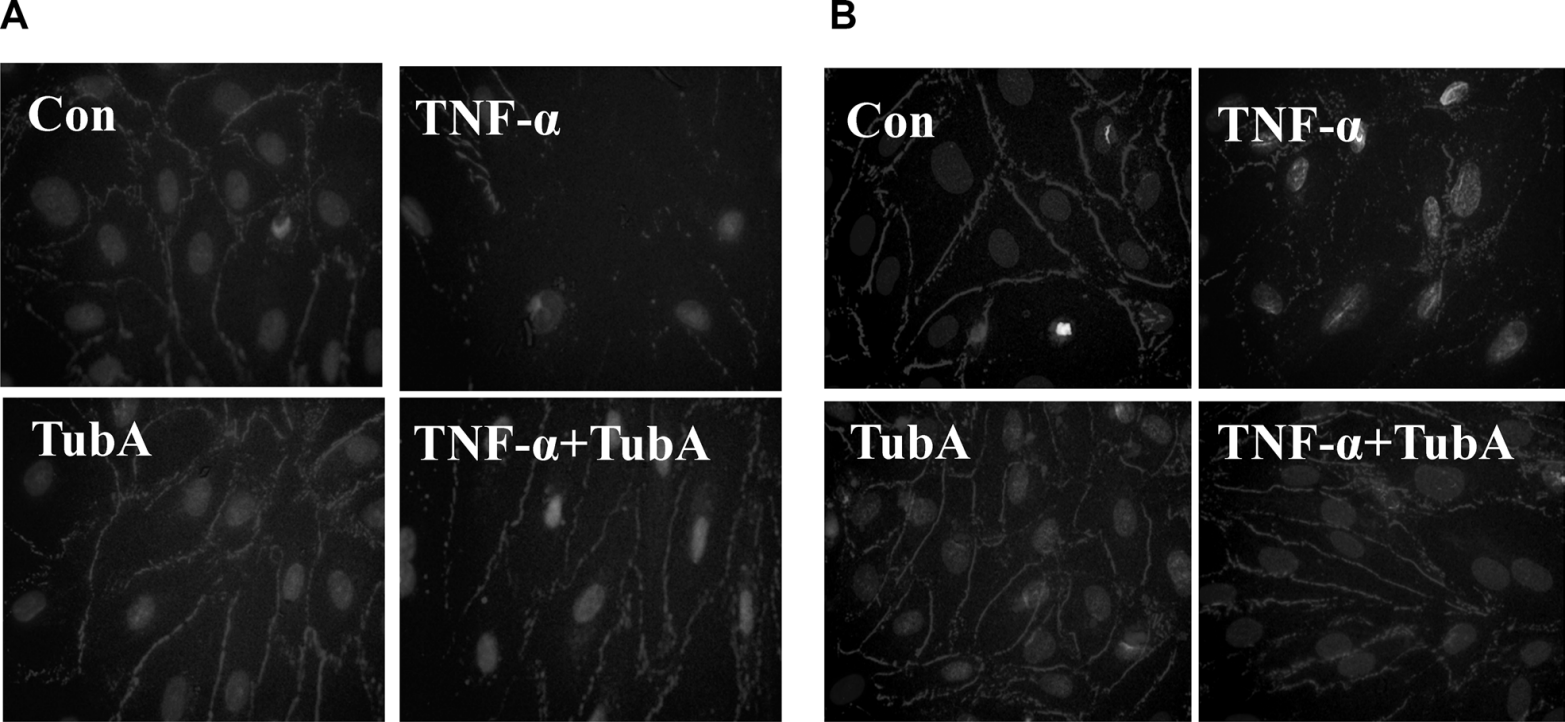
HDAC6 inhibition prevents TNF-α-induced ZO-1 disassembly in endothelial cells HPAECs and HLMVECs were pre-treated with Tubastatin A (TubA, 3 μM) for 6 h, then challenged with TNF-α (20 ng/ml) for 18 h. Cells were divided into 4 groups: Control (Con), TNF-α alone (TNFα), Tubastatin A alone (TubA), and TNF-α+Tubastatin A (TNFα+TubA). Immunofluorescence staining of ZO-1 in HPAECs (**A**) and HLMVECs (**B**). Images are representatives of three to six independent experiments.

Caspase-3 activation is associated with endothelial cell-cell junction disruption [2–4]. We next conduct experiments to assess whether HDAC6 inhibition could prevent TNF-α-induced damage to endothelial cell-cell junctions. We observed endothelial cells ZO-1 disassembly at tight junctions after TNF-α challenged by immunofluorescence assay. Pre-treatment with Tubstatin A prevented the disruption in HPAECs and HLMVECs (Figure 3).

### HDAC6 inhibition blocks endotoxin-induced caspase-3 activation and VE-Cadherin down-regulation in the lung

**Figure 4:**
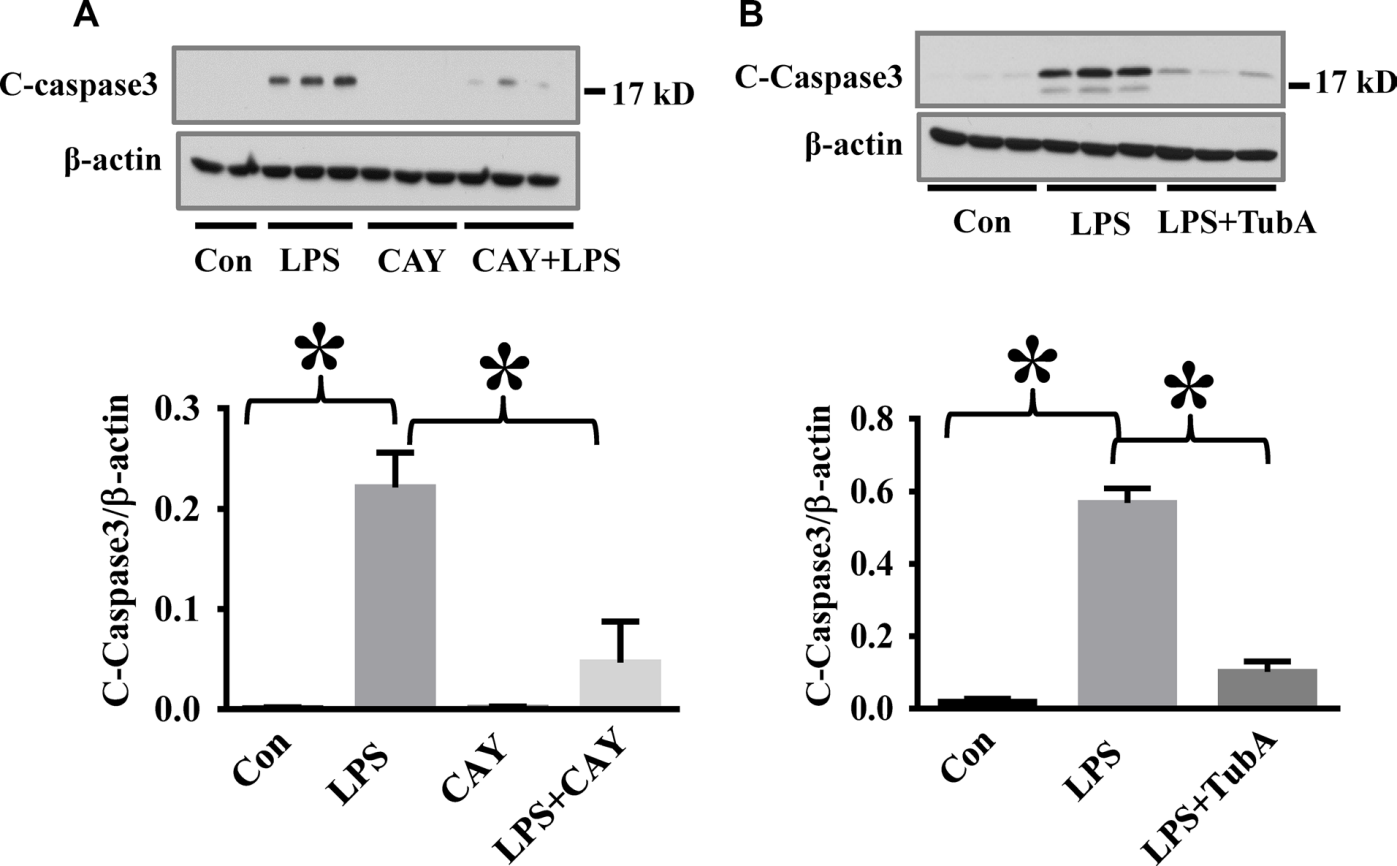
HDAC6 inhibition reduced LPS-induced lung caspase-3 activation in endotoxemia (**A**) C57BL/6 mice were divided into four groups: Control (Con, *n* = 6); LPS (LPS, *n* = 7), CAY10603 (CAY, *n* = 6); CAY10603+LPS (CAY+LPS, *n* = 7). Mice were pre-treated with CAY10603 for 2 h, then challenged with LPS for 24 h. Lung tissue were collected. (**B**) C57/B6 mice were divided into three groups: Control (Con, *n* = 6); LPS (*n* = 7); Tubastatin A (TubA) + LPS (*n* = 6). Mice were pre-treated with Tubastatin A for 6 h, then challenged with LPS. 24 h after LPS challenge, lung tissues were collected. Representative blots and densitometry analysis of cleaved caspase-3 in lung tissues. *P < 0.05 versus LPS group.

**Figure 5:**
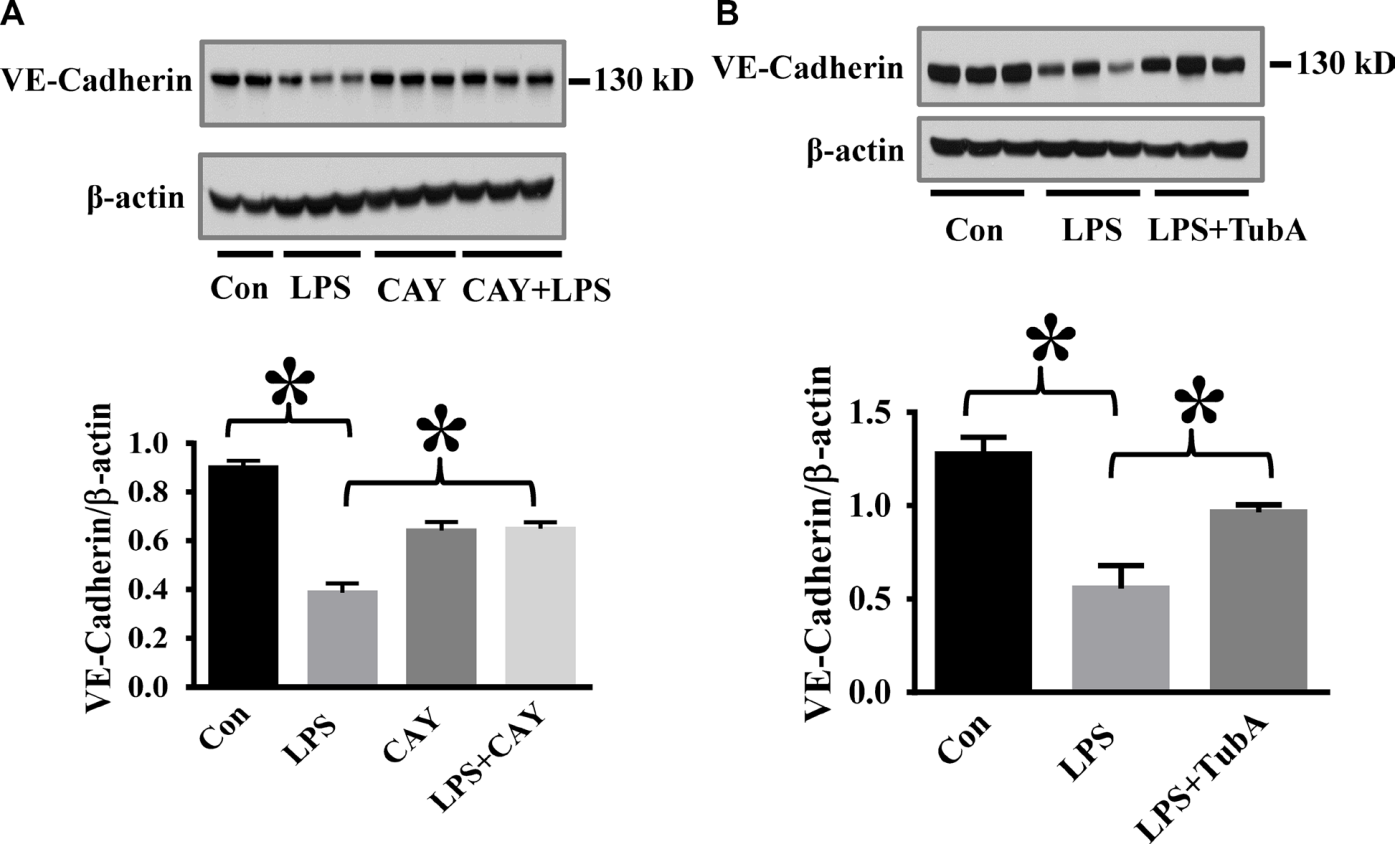
HDAC6 inhibition prevents LPS-induced VE-cadherin down-regulation in lung tissues (**A**) C57BL/6 mice were divided into four groups: Control (Con, *n* = 6); LPS (LPS, *n* = 7), CAY10603 (CAY, *n* = 6); CAY10603+LPS (CAY+LPS, *n* = 7). Mice were challenged by CAY10603 for 2 h, then challenged with LPS for 24 h. Lung tissue were collected. (**B**) C57BL/6 mice were divided into three groups: Control (Con, *n* = 6); LPS (*n* = 7); Tubastatin A (TubA)+LPS (*n* = 6). Mice were challenged by LPS with or without Tubastatin A (TubA) pre-treatment. 24 h after LPS challenge, lung tissues were collected. Representative blots and densitometry analysis of VE-cadherin level in lung tissues. *P < 0.05 versus LPS group.

To assess the therapeutic potential of HDAC6 inhibition against endothelial cell injury during acute inflammation, we examined the effects of CAY10603 on caspase-3 activation in a mouse model of endotoxemia. In our studies, endotoxin-induced caspase-3 activation in the lung tissues was significantly inhibited by CAY10603 and Tubastatin A pre-treatment. (Figure 4). Furthermore, endotoxin challenge caused down-regulation of adherens junction protein VE-Cadherin in the lung tissues. The down-regulation of VE-Cadherin was also blocked by CAY10603 and Tubastatin A pre-treatment (Figure 5).

### HDAC6 inhibition reduces TNF-α-induced endothelial permeability and attenuates lung edema formation in endotoxemia

**Figure 6:**
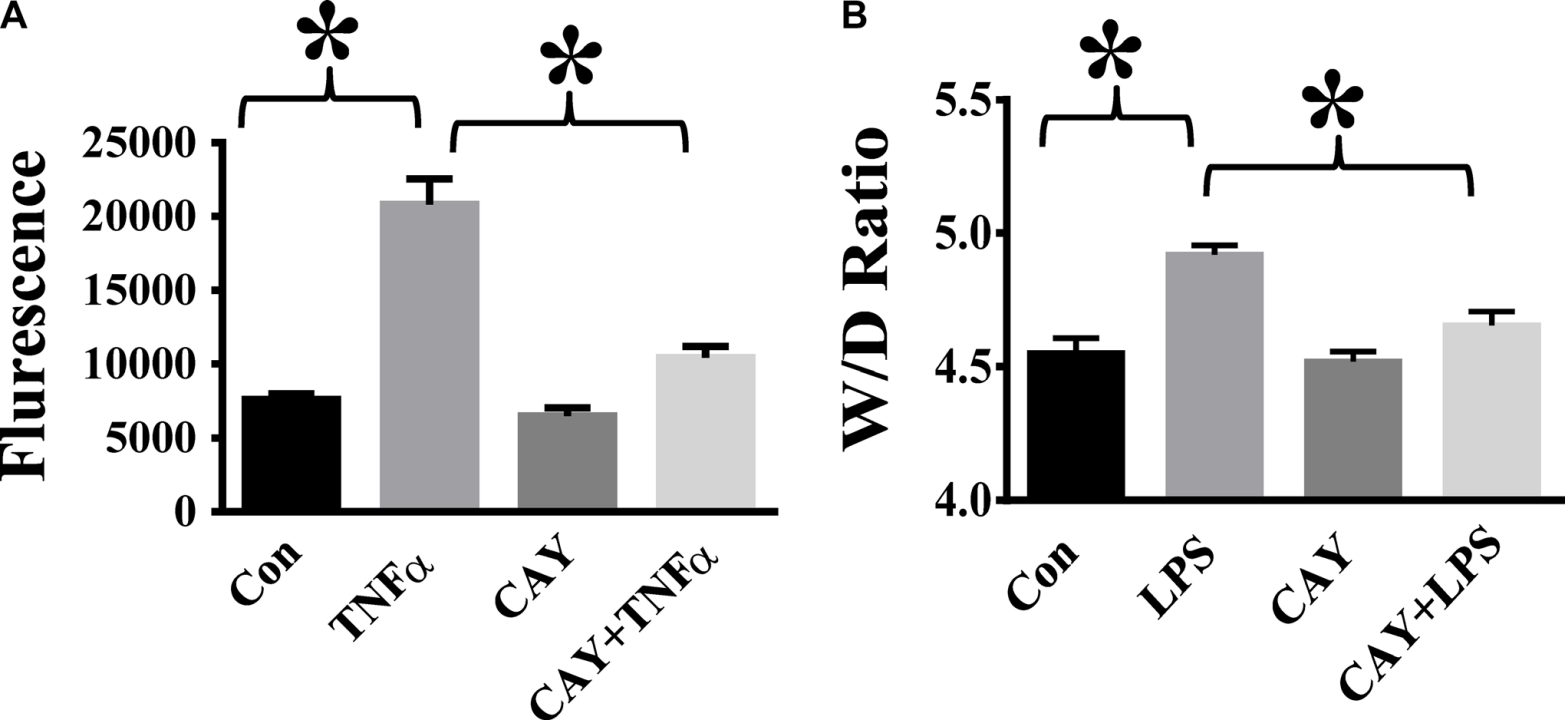
HDAC6 inhibition alleviates TNF-α-induced endothelial hyper-permeability and lung edema in endotoxemia (**A**) HPAECs were pre-treated with CAY10603 (CAY, 0.1 μM) for 6 h, then challenged with TNFα (20 ng/ml) for 18 h. Cells were divided into 4 groups: Control (Con), TNF-α alone (TNFα), CAY10603 alone (CAY), and TNF-α+CAY10603 (TNFα+CAY). TNF-α-induced endothelial permeability to FITC-Dextran was measured (*n* = 4). *P < 0.05 versus TNF-α group. (**B**) C57BL/6 mice were divided into four groups: Control (Con, *n*= 6); LPS (LPS, *n* = 7), CAY10603 (CAY, *n* = 6); CAY10603+LPS (CAY+LPS, *n* = 7). Mice were pre-treated with CAY10603 for 2 h, then challenged with LPS for 24 h. Lung Wet/Dry weight ratio was measured. *P < 0.05 versus LPS group.

TNF-α is a major pro-inflammatory mediator known to induce trans-endothelial hyper-permeability during inflammation [1, 7, 9]. We examined the effects of HDAC6 inhibition by CAY10603 on TNF-α-induced endothelial permeability. TNF-α challenge caused increased endothelial permeability to FITC-dextran in HPAECs. Cells pre-treated with CAY10603 exhibited significant reduction of TNF-α-induced endothelial permeability to FITC-dextran (Figure 6A). We also examined the effects of CAY10603 on lung vascular permeability in the mouse model of endotoxemia. In our studies, endotoxin-induced lung edema formation was significantly inhibited by CAY10603 pre-treatment (Figure 6B).

## DISCUSSION

TNF-α is a potent pro-inflammatory mediator known to cause endothelial cell apoptosis and endothelial barrier disruption [7, 9, 31–33]. TNF-α plays an important role in many inflammatory diseases including lung vascular injury [1]. Activation of caspases including caspase 3 contributes to TNF-α induces endothelial cell dysfunction such as hyper-permeability [2–4]. Caspase activation mediates apoptotic signaling and disruption of cell junction complex [2–4], contributing to TNF-α-induced breakdown of endothelial barrier function [2–4]. Similar effects were observed in our *in vitro* studies in primary human lung endothelial cells. Therefore, new agents that can inhibit TNF-α-induced caspase activation and preserve endothelial cell junctions could serve as therapeutic agents against TNF-α-induced endothelial cell dysfunction.

HDAC6 inhibition has been reported to prolong survival in murine models of systemic inflammation and injury [20, 24, 25]. However, mechanisms underlying the protective effects observed by HDAC6 inhibition remain to be determined. In the present study, we demonstrated that HDAC6 inhibition potently inhibited TNF-α-induced caspase 3 activation and endothelial barrier dysfunction, suggesting that HDAC6 inhibition could provide protection against inflammation-mediated endothelial cell injury.

Caspase activation mediates apoptosis and re-distribution of proteins that are known to modulate endothelial barrier function including ZO-1 and VE-Cadherin [2–4]. Protein acetylation can modulate caspase activation [2]. In our studies, HDAC6 inhibition by HDAC6 inhibitors or siRNA knockdown resulted in reduced caspase 3 activation. It has been reported that microtubule disruption leads to increased apoptotic signaling and caspase activation [34, 35]. α-tubulin is a endogenous substrate of HDAC6 [10, 11, 36–38]. Acetylation of α-tubulin at Lys40 stabilizes microtubule structures [12, 38–41]. The decreased caspase 3 activation by HDAC6 inhibition could be due to its protection against microtubule disassembly by α-tubulin acetylation. HDAC6 has been reported to modulate the function of chaperones such as Hsp90 [17]. Inhibition of HDAC6 down-regulates Hsp90-dependent RhoA activity and signaling, which then suppresses actin cytoskeleton re-organization and cell contraction [17]. HDAC6 has also been reported to modulate the function of Survivin, an anti-apoptotic protein [42]. Survivin inhibits apoptotic signaling by inactivating caspases [42]. Our data suggest that inhibition of caspase-3 activation by HDAC6 inhibition provides potent protection against TNF-α-induced endothelial cell dysfunction.

We also showed that HDAC6 inhibition effectively suppress caspase-3 activation and maintain lung endothelial barrier integrity *in vivo*. HDAC6 inhibitors prevented caspase 3 activation in lung tissues with reduced lung edema in a mouse model of endotoxemia. Maintaining VE-Cadherin expression is critical in supporting lung vascular barrier integrity [43]. We demonstrated that Tubastatin A and CAY10603 were able to prevent the reduction of VE-Cadherin in lung tissues after LPS challenge. Our results suggest that HDAC6 inhibition is a new approach that can modulate endothelial cell junction stability in inflammatory lung injury. More studies are needed in the future to further investigate this new pathway.

In summary, selective HDAC6 inhibition by CAY10603 and Tubastatin A prevents TNF-α-induced endothelial cell dysfunction. HDAC6 inhibitors prevent caspase-3 activation in endothelial cells and inhibit TNF-α-induced endothelial barrier dysfunction by maintaining cell-cell junction integrity. HDAC6 inhibitors prevented endotoxin-induced lung caspase-3 activation and lung edema, suggesting that selective HDAC6 inhibition possesses therapeutic potential to treat endothelial cell dysfunction during acute inflammation.

## MATERIALS AND METHODS

### Reagents

β-actin, ZO-1, caspase-3 antibodies were purchased from Cell Signaling Technology (Danvers, Massachusetts). VE-cadherin antibody was obtained from Santa Cruz Biotechnology (Dallas, Texas). Recombinant Human TNF-α was from R&D Systems (Minneapolis, Minnesota). Tubastatin A, CAY10603 were obtained from Selleck Chemicals (Houston, Teaxs). Lipopolysaccharide (LPS) from Escherichia coli 0111:B4 was purchased from Sigma Aldrich (St. Louis, Missouri). Endothelial growth medium (EBM-2) was obtained from Lonza (Allendale, NJ). *In Vitro* Vascular Permeability Assay (96-Well) kit was purchased from EMD Millipore (Billerica, Massachusetts). Lipofectamine RNAiMAX reagent, HDAC6 siRNA and control siRNA were obtained from Life technologies (Carlsbad, California).

### Cell culture

Human pulmonary arterial endothelial cells (HPAECs) and human lung microvascular endothelial cells (HLMVECs) were purchased from Lonza (Allendale, NJ). Cells were grown in EGM-2 supplemented with fetal bovine serum (FBS) and cultured in an incubator at 37°C in 5% CO2 and 95% air. Cells from passages 5 to 9 were used in the experiments.

### HDAC6 siRNA knockdown

HDAC6 siRNA knockdown was conducted as described previously [41]. Cells were transfected with siRNAs for 48 h according to the manufacture's protocol. siRNA-transfected cells were then stimulated with 20 ng/ml TNF-α for 24 h.

### Immunofluorescence and immunoblotting assays

For immunofluorescence assay, cells were grown on glass coverslips pre-coated with 0.1% gelation. After the treatment, cells were fixed in −20°C methanol and washed with PBS on ice. Cells were blocked with 1% BSA in PBS, then incubated with primary antibody. After 3 washes with TBS, cells were incubated with Alexa Fluor 594 secondary antibody. The coverslips were mounted on the slides with DAPI. Immunoblotting assays of cell and lung tissue samples were conducted as described previously [43, 44].

### Mouse model of endotoxemia

Eleven to twelve week-old male C57BL/6 mice were purchased from Jackson Lab. All experiments and animal care procedures were approved by the Institutional Animal Care and Use Committee of the University of Kentucky. Endotoxemia was induced by I.P. injection of 7.5 mg/kg LPS in phosphate buffered saline (PBS). Mice were pre-treated by I.P. injection with Tubstatin A (9 mg/kg body weight) for 6 h or CAY10603 (5 mg/kg body weight) for 2 h before LPS challenge. Experiments were terminated 24 h after LPS challenge. Lung wet/dry weight ratio was assessed as described previously [43].

### Endothelial cell permeability assay

*In Vitro* Vascular Permeability Assay (96-Well) kit was used to measure endothelial cell monolayer permeability to FITC-dextran according to the manufacture's protocol. Briefly, endothelial cells were seeded into the insert. 72 h later, endothelial cells formed a monolayer. Endothelial cell monolayers were pre-treated with CAY10603 (CAY, 0.1 μM) for 6 h, and then stimulated with TNF-α for 18 h. A high molecular weight (2000 kD) FITC-Dextran was diluted in EGM-2 medium (1:40) and added into the insert. 20 minutes after the incubation, 100 μl medium from lower chamber was collected. Fluorescence intensity was measured in a plate with a fluorescence plate reader.

### Statistical analysis

Results are expressed as means ± SE of 3 to 6 independent experiments. ANOVA and post hoc multiple comparison tests were used for multiple groups. The Student's *t-test* was used for comparisons of two groups. Statistical significance was assigned to *P* values < 0.05.
